# Lipid–protein forces predict conformational changes in a mechanosensitive channel

**DOI:** 10.1007/s00249-020-01488-z

**Published:** 2020-12-23

**Authors:** Csaba Daday, Bert L. de Groot

**Affiliations:** Department of Theoretical and Computational Biophysics, Computational Biomolecular Dynamics Group, Max Planck Institute for Biophysical Chemistry, Göttingen, Germany

**Keywords:** Membrane tension, Conformational change, Ion channel, Force distribution analysis, Functional mode analysis

## Abstract

The mechanosensitive TREK-2 potassium channel, a member of the K2P family, has essential physiological roles and is, therefore, a pharmaceutical target. A combination of experimental and computational studies have established that of the two known conformations, “up” and “down”, membrane tension directly favors the “up” state, which displays a higher conductance. However, these studies did not reveal the exact mechanism by which the membrane affects the channel conformation. In this work, we show that changes in protein–lipid interaction patterns suffice in predicting this conformational change, and pinpoint potentially important residues involved in this phenomenon.

**Supplementary Information:**

The online version contains supplementary material available at 10.1007/s00249-020-01488-z.

## Introduction

The family of two-pore-domain potassium channels (the K2P family) contains 15 members and are generally inward-rectifying channels contributing to a leak current and are characterized by a unique dimer structure. They are attractive drug targets, having been linked to diseases as wide-ranging as hypertension (Lloyd et al. 2011) to depression (Gotter 2011). Despite the fact that these proteins do not have fourfold symmetry like most other potassium channels, the selectivity filter is very similar to the canonical one, with four strands forming potassium binding sites through their backbone carbonyl groups (Braun 2012; Feliciangeli et al. 2015).

Of the K2P family, three members are known to be activated by mechanical force: TREK-1 (K2P2), TREK-2 (K2P10), and TRAAK (K2P4) (Brohawn et al. 2014). These mechanosensitive proteins have roles in a wide range of physiological processes such as the sense of touch and pain perception (Plant 2012), and for this reason, a wide range of studies has been performed on them. Stimuli such as temperature and pH have been identified as also regulating them, and several drugs were found to bind to these channels, indicating their pharmacological role (Luo 2017; Djillani et al. 2019). Interestingly, these three proteins are so similar in structure that artificial heterodimers formed by them are also functional. (Blin 2016).

X-ray crystallography has identified two different conformational states of TRAAK and TREK-2, called “up” and “down” (Brohawn et al. 2012; Dong 2015), named after the orientation of the lower helix bundle. Initially, there was conflicting evidence about what the physiological role of these two conformations would be: the up state was shown to have higher ion occupancy (Brohawn et al. 2012; Zhou et al. 2001; Köpfer 2014), therefore, presumably more conductive, whereas the down state was thought to be more conductive based on mutational study (Lolicato et al. 2014).

In a recent computational study (Aryal 2017), the conformational change from “down” to “up” due to membrane tension in TREK-2 was captured, suggesting higher conductance of the up state. Later, this was directly confirmed (Brennecke and Groot 2018) using computational electrophysiology: the up state was found to exhibit higher conductance than the down state, and membrane tension induced, partially or fully, a conformational transition from the down to the up state. In fact, in the presence of membrane tension, the system had a higher conductance regardless of the starting structure. This later study also linked the change in conductance to a carbonyl flip in the selectivity filter, which was found to more likely occur in the down state and less likely in the presence of the membrane tension.

This forms an overall picture on the effect of membrane tension on channel conductance, but leaves unanswered how the signal propagates from the membrane to the protein. First, while it appears (Brohawn et al. 2014) that the membrane tension predominantly triggers the down/up conformational change, the nature of the involved lipid–protein interaction remains unknown. Second, the selectivity filter is several nanometers from the conformational change and it is thus unclear how the conformational change affects carbonyl flips in the selectivity filter. In this work, we will address the first question by an in-depth analysis of protein–lipid forces and their effect on the protein conformation.

Force distribution analysis (Stacklies et al. 2011; Costescu and Gräter 2013) (FDA) is a computational tool that traces pairwise forces in molecular dynamics (MD) simulations and identifies changes therein due to external perturbations. While one of the first applications of FDA was on a graphene sheet (Costescu and Gräter 2014), it is typically used on biomolecules, where it has successfully identified key residues in the force response of proteins (Aponte-Santamaría 2017; Butera, et al. 2018) Recently, FDA was also employed on lipid bilayers in two applications: first, it was used to quantify pulse propagation through a membrane (Aponte-Santamaría et al. 2017) and to assess force transmission in complex membrane (Ray et al. 2018). Here, we will use FDA to assess changes in lipid–protein interactions to understand how they induce the above-mentioned conformational changes in TREK-2. Specifically, we will combine FDA with partial-least-squares functional mode analysis (Hub et al. 2010; Krivobokova et al. 2012) (PLS-FMA) to address the question of how lipid–protein forces direct the down-up conformational change by identifying which contributions of residues in contact with the membrane are key in propagating the signal from the membrane to the protein.

## Methods

In our study, we perform ten equilibrium simulations and ten simulations under surface tension. To capture the previously obtained conformational structure, we chose to start the simulations of the “down” state (PDB: 4XDJ Dong 2015) of TREK-2 equilibrated previously (Brennecke and Groot 2018) and the CHARMM36 (Klauda 2010) force field combined with the TIP3P (Jorgensen et al. 1983) water model in GROMACS, version 2019 (Spoel 2005). The membrane was composed of 278 POPC lipids: 144 in the upper leaflet, 134 in the lower leaflet, corresponding to a uniform area per lipid.

Long-range electrostatic interactions were modelled using a fourth-order Particle Mesh Ewald method (Essmann 1995) with a grid spacing of 0.12 nm. The short-range electrostatic interactions had a cutoff of 1.2 nm, while the Lennard–Jones interaction was smoothly turned off between 1.0 and 1.2 nm. Three separate Nosé-Hoover thermostats with a chain length of ten kept the temperature at 300 K for the protein, the membrane, and the remaining atoms, respectively.

The pressure coupling was implemented through the Berendsen “surface tension” option in GROMACS, either at 0.4 mN/m or at 50 mN/m, which were the two extreme surface tensions in the previous study. A total of approximately 6 µs was simulated in the presence of surface tension and 6 µs in equilibrium as control trajectories. We applied no electrostatic potential in any of the simulations. We extracted frames with a time window of 40 ps for further analysis.

Force distribution analysis (FDA) was performed on the (non-bonded) interactions between lipids and the protein, and only the “punctual stress”, defined as the scalar sum of all forces between a given residue and all lipid molecules, was evaluated:

$$ {\text{PS}}_{i} = \sum\limits_{j}^{{N_{{{\text{lip}}}} }} {\left| {{\mathbf{F}}_{ij} } \right|} , $$

where *i* refers to a protein residue and *j* refers to a lipid. Only the short-range forces are included in this sum, and **F**_*ij*_ refers to the vector sum of the forces between all relevant pairs of atoms in the protein residue and the lipid, respectively. We used FDA version 2.7. FDA code is available at http://github.com/HITS-MBM/gromacs-fda.

Each extracted frame was, therefore, represented as a 521-dimensional vector, each one representing one residue.

As a measure for the conformational transition, we used the difference vector, defined as the projection onto the “principal component” between the “down” an “up” crystal structures, using backbone atoms only. Its functional form is given by

$$ x(t) = \sum\limits_{i}^{{N_{{{\text{BB}}}} }} {\left( {{\mathbf{r}}_{i} (t) - {\mathbf{r}}_{i}^{{{\text{avg}}}} } \right) \cdot \left( {{\mathbf{r}}_{i}^{{{\text{up}}}} - {\mathbf{r}}_{i}^{{{\text{down}}}} } \right),} $$

where

$$ {\mathbf{r}}_{i}^{{{\text{avg}}}} = \frac{1}{2}\left( {{\mathbf{r}}_{i}^{{{\text{up}}}} + {\mathbf{r}}_{i}^{{{\text{down}}}} } \right), $$

*i* loops through all backbone atoms, and all structures are fitted to the reference structure (the “down” state). This classification is performed for every analyzed frame in a trajectory.

To explain the conformational transition with the 521-dimensional vectors of residue-membrane forces, we use PLS-FMA. This is a method which performs multidimensional regression with built-in covariance optimization. Unlike principal-component analysis (PCA), PLS-FMA iteratively optimizes a basis that shows a maximal covariance with the observable it seeks to explain. Further details on its implementation can be found in Krivobokova et al. (Krivobokova et al. 2012). We perform tenfold cross-validation, that is, we run PLS-FMA on our data set ten times, in each case holding out one trajectory for validation.

The default version of PLS-FMA, running on 3D coordinates, is available at http://www3.mpibpc.mpg.de/groups/de_groot/fma.html. The modified code, running on arbitrary input (in this case, membrane-residue forces), is available upon request.

## Results

**Fig. 1:**
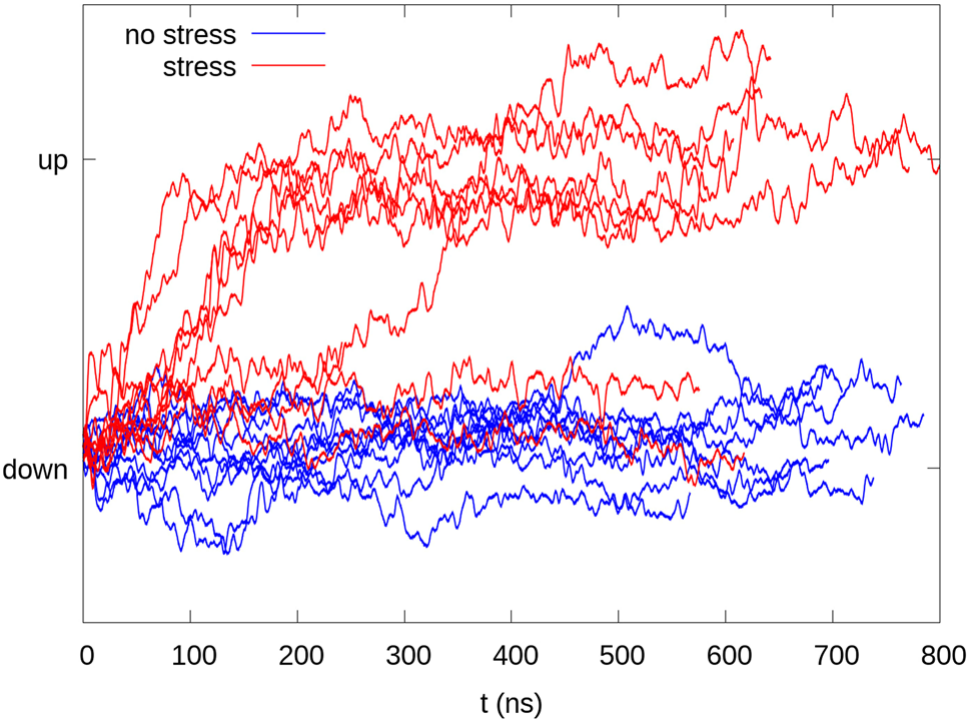
Conformational change in TREK-2 under tension. The ten simulations with tension are traced in red and the ten control simulations without tension in blue. The difference vector (labelled by the two crystal structures) shows the conformational change in 7/10 replicas

In 7 out of the 10 replicas with surface tension, we observe a clear down-to-up transition, as defined by the difference vector between the up and down x-ray conformations, as defined previously. As can be seen in Fig. 1, the transition occurs after approximately 50–300 ns in the trajectories. In contrast, in the control simulations performed without any tension, we observe only minor fluctuations close to the down state. This is in agreement with our previous work (Brennecke and Groot 2018).

**Fig. 2:**
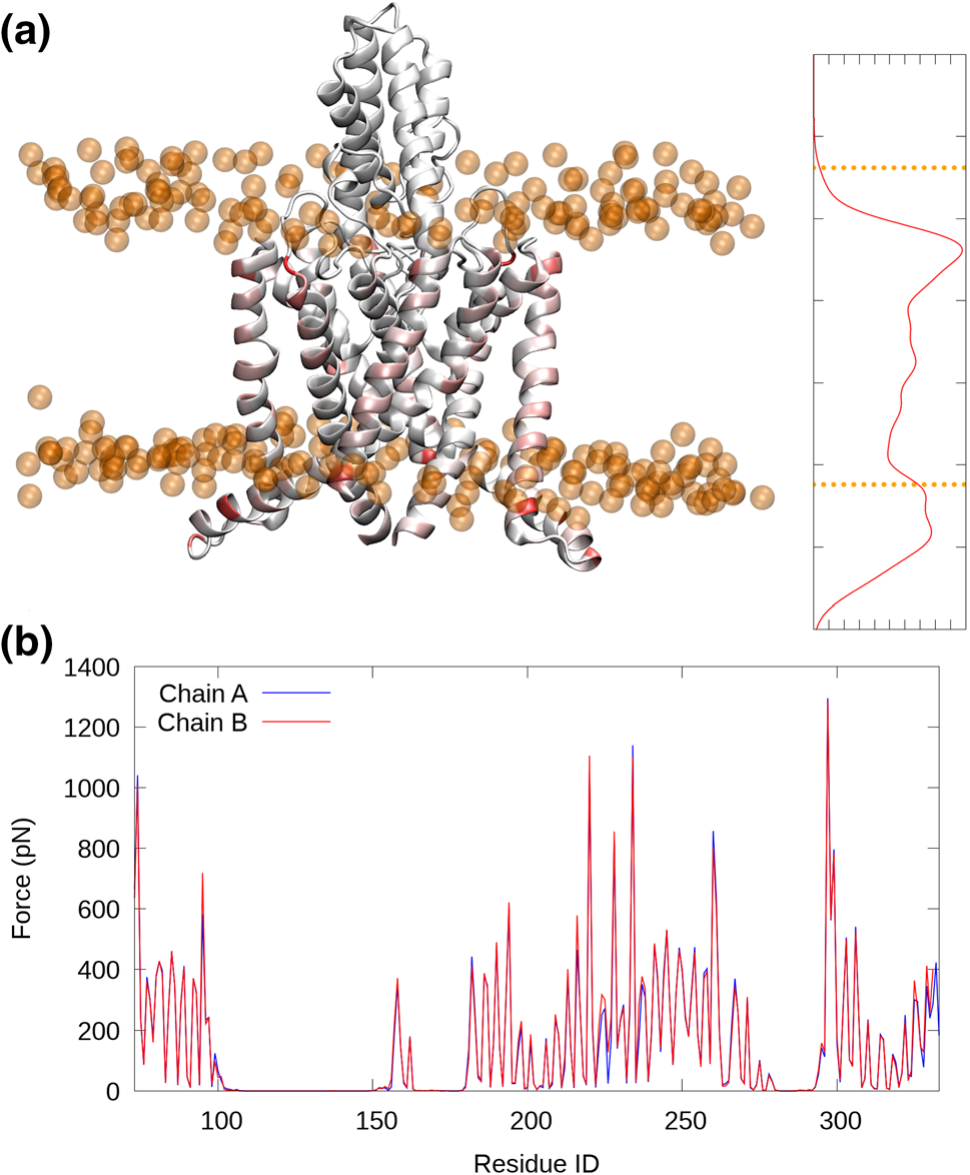
Stress transmission concentrates close to the head groups. **a** 3D structure of TREK-2 is color-coded as a function of protein–lipid interaction with an approximate overlay of the stress profile projected onto the *z* axis. The phosphorus atoms of the POPC head groups are shown as orange spheres. **b** Protein–lipid interaction has a very similar profile across the two dimers

To understand the protein–lipid interactions underlying this conformational change, we employ force-distribution analysis and consider only the interactions between the protein residues and the lipids, with the aim to identify residues that are particularly affected by membrane tension and thus may play a key role in facilitating the conformational transition. We concentrate only on the simulations under surface tension as they are the group of simulations which exhibited the conformational change. As a first exploratory analysis, we consider all of the trajectories (before and after the conformational change). As can be seen in Fig. 2a, most residues exposed to lipid tail groups experience forces from the lipids, but certain specific residues close to the head groups are exposed to particularly high forces. Since the channel is formed by a dimer, we can compare the data from the two chains and assess the deviation from perfect symmetry as a proxy for convergence. We can see (Fig. 2b) that the stress profiles are very consistent, meaning that the signal is most likely statistically significant and not only due to thermal fluctuations or other sources of noise.

For a more fine-grained analysis, we now turn to understanding how these forces change due to the conformational change. In particular, we set out to answer the question if the protein conformational change can be predicted solely from protein–lipid forces. To achieve this, we applied PLS-FMA with the protein–lipid forces as input coordinates and the up-down difference vector as target variable. To minimize the danger of overfitting, we perform cross-validation by holding out each replica as a validation set successively (complete cross-validation). In all 7 test cases, where a transition has taken place, our PLS vector predicts the transition with a receiver operating characteristic curve (ROC) area under curve (AUC) between 0.94 and 0.99 (see SI for the full data set) and a correlation between predicted and observed values of the difference vector between 0.70 and 0.89. For the three trajectories where no transition was observed, the model gives 0.2%, 23%, and 58% false positives (falsely predicting a transition). In summary, our PLS model performs well on 9 out of 10 possible validation sets.

The obtained PLS vector is very similar across the ten possible training data sets (see SI) and the contribution of the ensemble-weighted vector (the first PLS eigenvector) is between 67 and 70%, meaning that a single, dominant collective set of protein–lipid forces forms the major contribution to the enforced conformational change. For further analysis, we will use the average of the ten such-obtained PLS vectors.

**Fig. 3:**
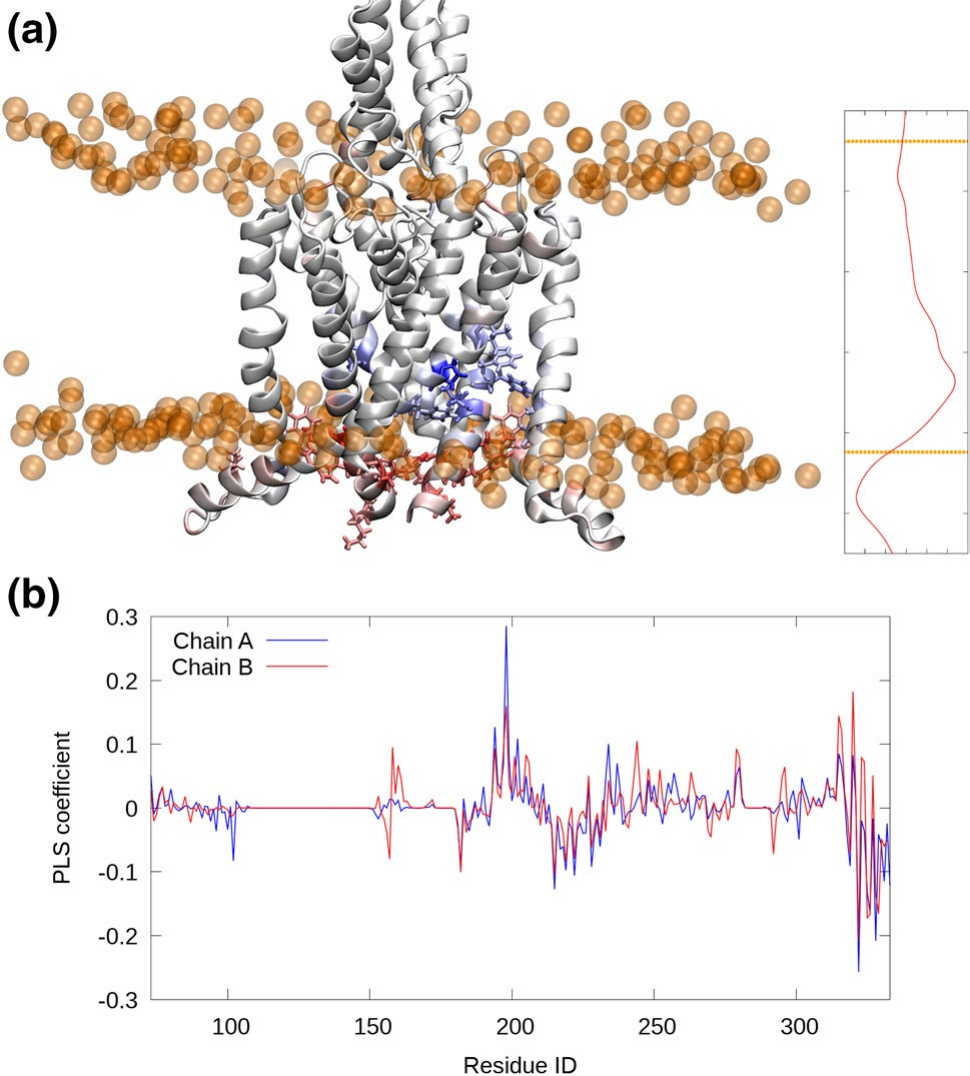
‘Force hotspots’ are concentrated at the lower leaflet. The residues are colored according to the PLS vector coefficients (**a**) and shown as coefficients from the two chains separately (**b**), displaying a substantial level of correspondence

Figure 3 projects the PLS vector onto the 3D structure of the protein. Not surprisingly, since the transition occurs in the lower helix bundle, the PLS vector has large contributions in this region, and similarly to the initial analysis on the forces only, we see that the most important residues are the ones close to the head groups. The residues above the head groups have a positive component, meaning larger stress in the ‘down’ state, and those below the head groups a negative component, larger stress after the transition to the ‘up’ state.

The PLS vector has similar coefficients across both chains (Fig. 3b), but also deviations. As the channel is a two-fold symmetric dimer, the observed differences between the A and B chains are likely the result of statistical noise, and therefore, we rather focus on common features observed for both chains. For example, the most positive coefficient is observed for P198 in Chain A (0.285), whose corresponding residue in Chain B is the third-most positive coefficient (0.159). Similarly, the most negative coefficient is M322 in Chain A, and the same residue in Chain B has the third-most negative coefficient. A full comparison of Chains A and B can be found in the Supporting Information (Tables S2a,b and Figure S2). A perusal of the raw data (Figure S4) indeed shows that P198 feels higher forces from the membrane before the transition and M322 feels higher forces after the transition.

In a multiple sequence alignment of K2P channels (Figure S5), P198 is highly conserved (13/15 = 87%), whereas M322 is only present in TREK-2 and the related mechanosensitive channel TREK-1. It thus appears that the conservation pattern of these residues may be connected to their role in mechanosensing but the correspondence is not strict, rendering it likely that also other factors contribute. In agreement with our findings, the M322A mutant is already known to show less stretch activation (Dong 2015) and the precise role M322 has in stabilizing the “up” state was discussed elsewhere (Aryal 2017). In total, 4 of the 10 identified residues (5 most positive and 5 most negative average coefficients) have been experimentally tested as alanine mutants, and all of them showed reduced stretch activation (see Tables S2a,b). P198, on the other hand, is a “fenestrating residue” involved in binding the drug BL-12 (Schewe 2019). However, it is unclear how this role relates to the force activation described in this work.

## Discussion

In this work, we presented a predictive model of a conformational transition in a membrane protein using only information from forces exerted by the membrane. This is remarkable as it not only underscores that the up/down transition in the TREK-2 channel is indeed induced by membrane tension, but also allows pinpointing force hotspots critically involved in the transition, which, therefore, provide insight in the underlying mechanism. Similar methods could hold promise for a wide range of systems responsive to mechanical stress.

By comparing the time evolution of the forces with the conformational changes from the “down” to the “up” state of the mechanosensitive K2P channel TREK-2, we found that after the transition, residues embedded in the lower leaflet experience a drop in force, while those closer to the head groups have stronger interactions with lipids. We also identified the residues most predictive of the transitions: P198 and M322.

There are two different plausible interpretations of our data: either the identified “force-sensing residues” are key to the mechanism, or it is a more collective phenomenon involving the structure of the protein, and hence the position of the involved residues, rather than the type of amino acid at these positions. The fact that M322A has been independently verified as a variant with reduced stress response would seem to argue for the first picture.

We validated the linear combination of forces identified by PLS-FMA in two ways: first, by cross-validation through independent replicas and second, by comparing the coefficients obtained on the two chains of the symmetric dimer. Our model showed good predictive power during cross-validation and had a consistent set of coefficients in the two chains, in line with the symmetry of the structure. It should be noted that a perfect level of symmetry between the two chains cannot be expected. First, the trajectories are stochastic, rendering the force signal noisy. This statistical noise cannot be expected to fully average out in the limited statistics of the ten trajectories at hand. Second, although the starting X-ray conformation is fully symmetric, the individual MD snapshots are not. Therefore, channels undergoing the down-to-up transition end up in partially asymmetric states. This asymmetry will also be reflected in the lipid–protein forces used by PLS-FMA.

Finally, we note that while the majority of our trajectories (90%) showed a single transition pathway, we observed one outlier trajectory in which the protein–lipid forces changed without a conformational transition. This suggests that there are alternative mechanisms that can change protein-membrane interactions. More sampling would be necessary to further understand these mechanisms, but with our given data and the partial experimental validation, we already have a consistent picture of how the conformational change is triggered when it is triggered in the majority of the cases.

In this study we identified a well-defined protein–lipid interface for force transmission that provides a mechanistic picture of how membrane stress is propagated to the protein in the form of a conformational change. It will be interesting to analyse how this contributes to the overall allosteric mechanism. In particular, how does the signal propagate from this interface to the selectivity filter, and how does it affect the previously observed carbonyl flips in the selectivity filter, that underlie changes in ion permeation rates? Further studies would be required for a complete picture of force-induced change in conductance.

## Supplementary Information

Below is the link to the electronic supplementary material.

Supplementary file1 (DOCX 1413 KB)
