# Supplementary material for: Lipid–protein forces predict conformational changes in a mechanosensitive channel
Source: Eur Biophys J. 2020 Dec 23;50(2):181–6. doi: 10.1007/s00249-020-01488-z (PMC8071793; doi:10.1007/s00249-020-01488-z)
Supplement: Supplementary file 1 — Supplementary file1 (DOCX 1413 KB) [file 249_2020_1488_MOESM1_ESM.docx]

**Supplementary Material for:**

**Lipid-Protein Forces Predict Conformational Changes in a Mechanosensitive Channel**

Csaba Daday^1^, Bert de Groot^1,*^

^1^Department of Theoretical and Computational Biophysics, Computational Biomolecular Dynamics Group, Max Planck Institute for Biophysical Chemistry, Göttingen, Germany

*bgroot@gwdg.de

| Val | FPR | FNR | AUROC | r | EW weight |
| --- | --- | --- | --- | --- | --- |
| 1* | 0.23 | - | - | 0.66 | 0.68 |
| 2* | 0.003 | - | - | 0.13 | 0.67 |
| 3* | 0.58 | - | - | 0.18 | 0.68 |
| 4 | 0.17 | 0.02 | 0.988 | 0.86 | 0.68 |
| 5 | 0.24 | 0.02 | 0.985 | 0.82 | 0.68 |
| 6 | 0.25 | 0.08 | 0.943 | 0.84 | 0.67 |
| 7 | 0.08 | 0.02 | 0.993 | 0.82 | 0.67 |
| 8 | 0.14 | 0.04 | 0.982 | 0.89 | 0.68 |
| 9 | 0.02 | 0.05 | 0.994 | 0.70 | 0.70 |
| 10 | 0.28 | 0.01 | 0.956 | 0.76 | 0.69 |

**Supplementary Table S1**: detailed evaluation of the 10 validation sets. We give false positive rates (FPR), false negative rates (FNR), the area under the receiver-operator curve (AUROC), the Pearson correlation between predicted and observed difference vector values (r), and the ensemble-weighted vector weight (EW weight), as a function of the replica used for validation (Val). Trajectories 1-3 show no transitions, therefore false negatives and ROC analysis are impossible.


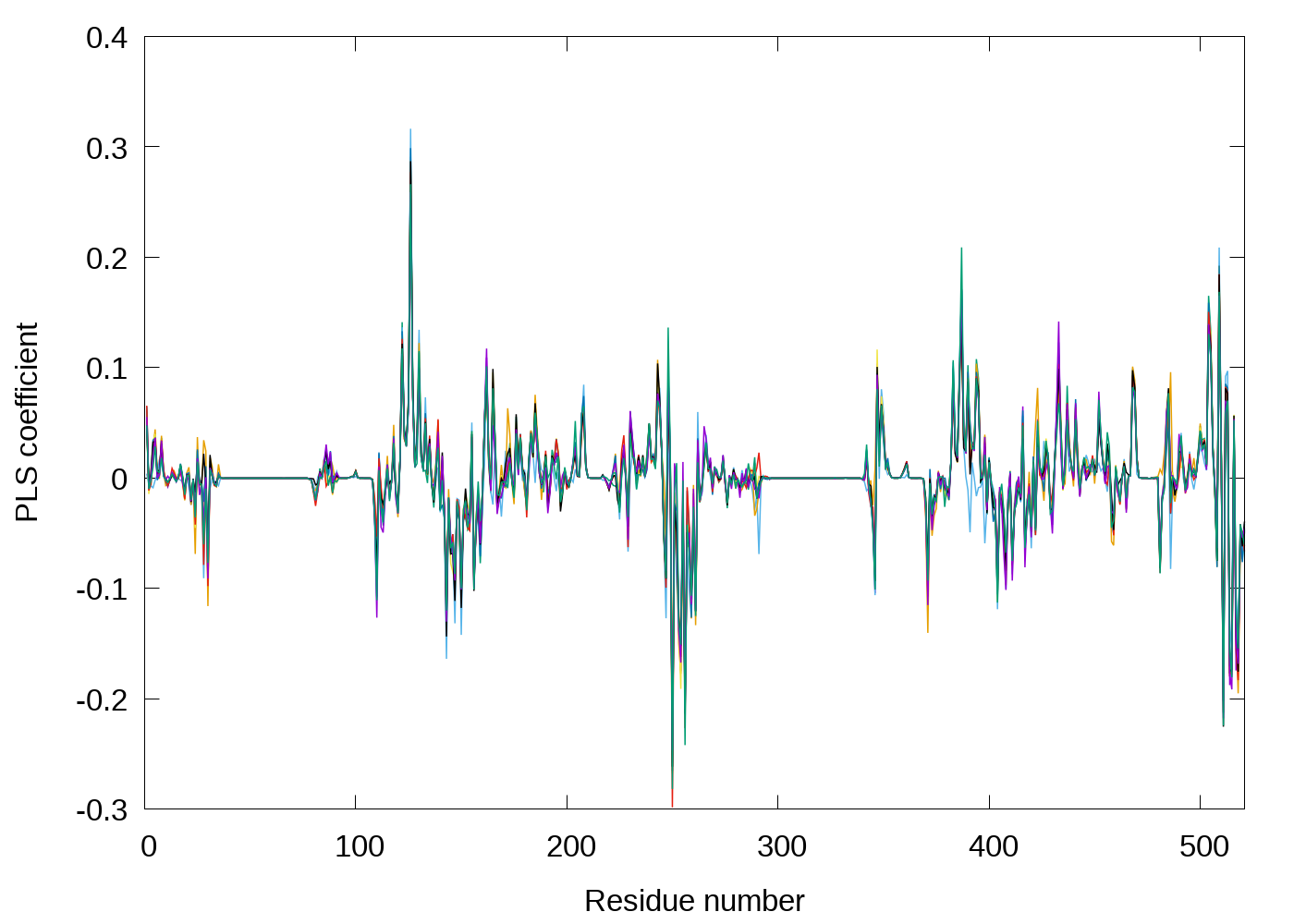


**Supplementary Figure S1**: the coefficients of all 10 obtained PLS vectors.

| Residue | Chain A | Chain B | Rank A | Rank B | Rank A+B |
| --- | --- | --- | --- | --- | --- |
| P198 | 0.285 | 0.160 | 1 | 2 | 1 |
| L320 | 0.083 | 0.182 | 6 | 1 | 2 |
| **Y315** | 0.085 | 0.144 | 5 | 3 | 3 |
| I194 | 0.127 | 0.094 | 2 | 7 | 4 |
| F316 | 0.065 | 0.120 | 10 | 4 | 5 |

| Residue | Chain A | Chain B | Rank A | Rank B | Rank A+B |
| --- | --- | --- | --- | --- | --- |
| **M322** | -0.257 | -0.208 | 1 | 1 | 1 |
| R328 | -0.208 | -0.135 | 2 | 5 | 2 |
| **W326** | -0.163 | -0.166 | 3 | 3 | 3 |
| D325 | -0.132 | -0.172 | 4 | 2 | 4 |
| **F215** | -0.127 | -0.105 | 5 | 6 | 5 |

**Supplementary Tables S2a,b**: The top 5 most positive (top) and most negative (bottom) coefficients in the PLS vector. “Rank A/B” refer to the ranking of that residue in chain A/B, while “Rank A+B” to the ranking of the sum of coefficients. In bold, four residues whose alanine mutants have been shown to reduce activation by stretch in Dong et al 2015.


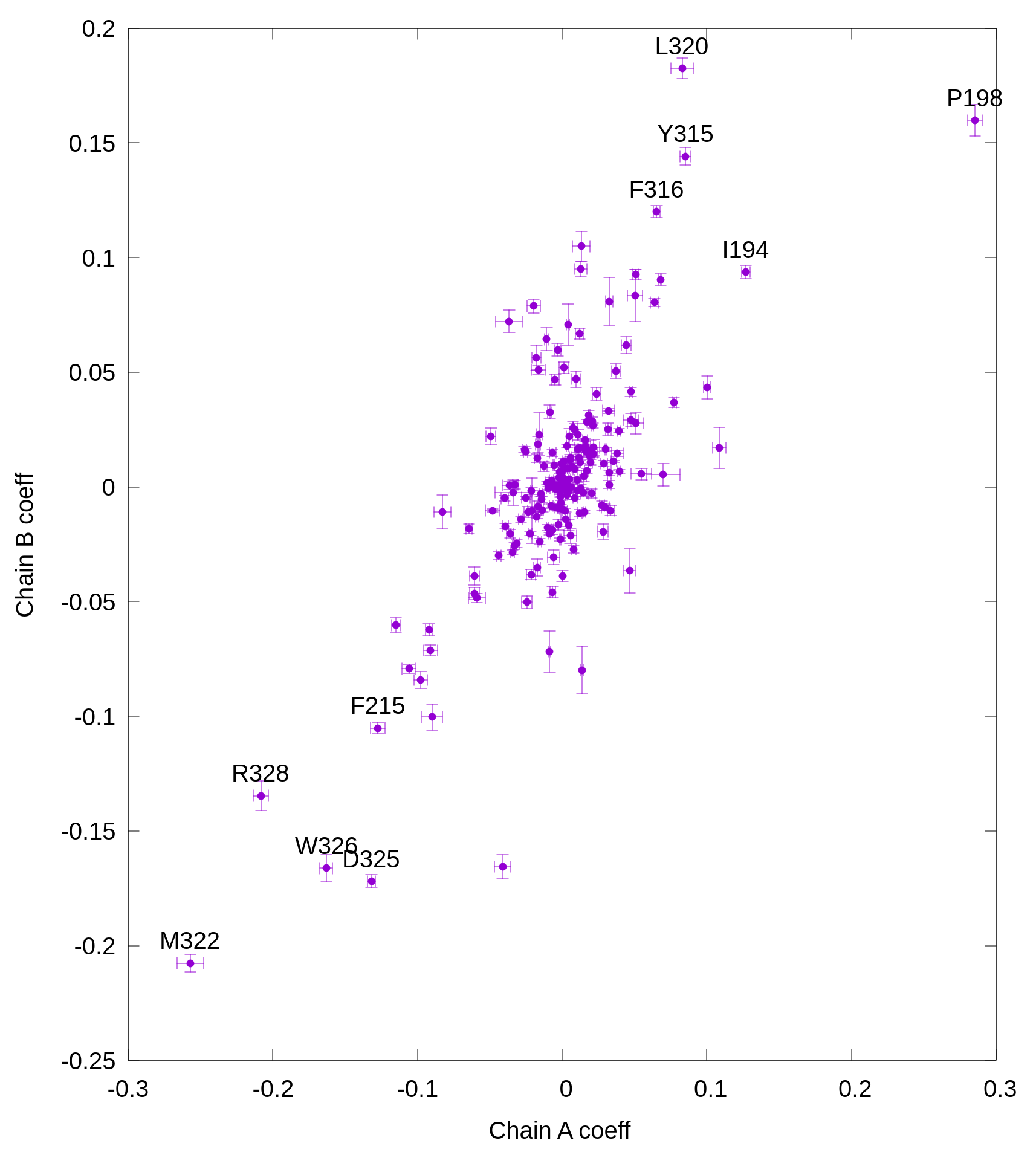


**Supplementary Figure S2**: The comparison of the coefficients of the same residues in Chain A and Chain B. The standard error of the mean across the 10 obtained models is shown. Only the highest and lowest 5 coefficients (by the sum of the two chains) are labeled by residue.


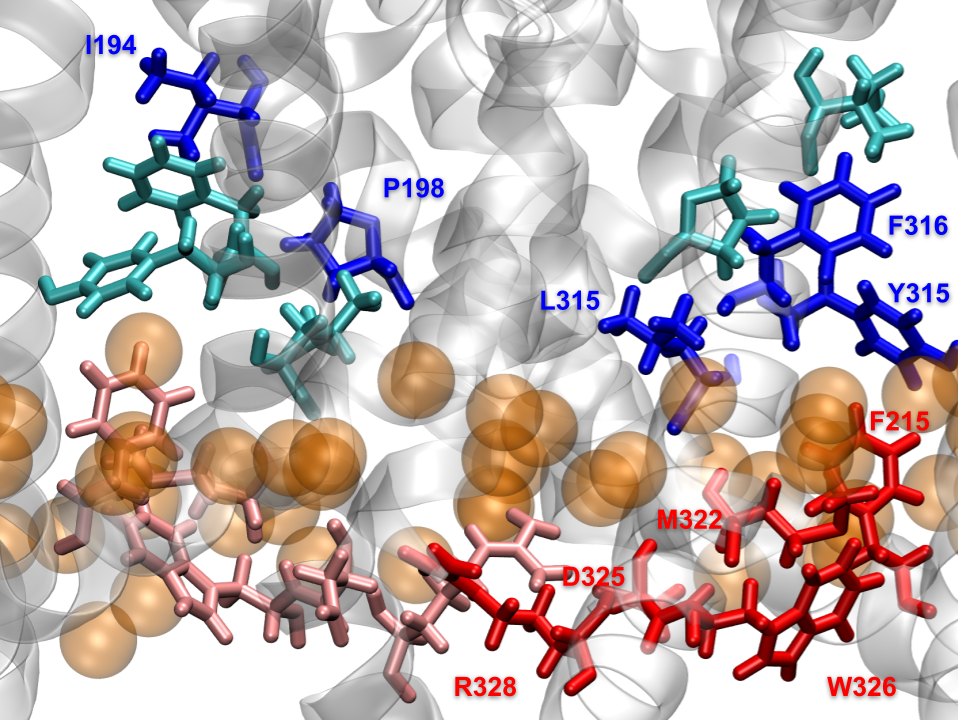


**Supplementary Figure S3**: The position of the top 5 highest/lowest coefficients. The phosphorus atoms of the head groups of the leaflets can be seen as orange spheres. Only the residues of chain A (in dark blue and red) are labelled, whereas the residues of chain B are represented in cyan and pink. Blue and cyan residues correspond to positive coefficients (higher forces before the transition), and red and pink residues correspond to negative ones (higher forces after the transition).

**
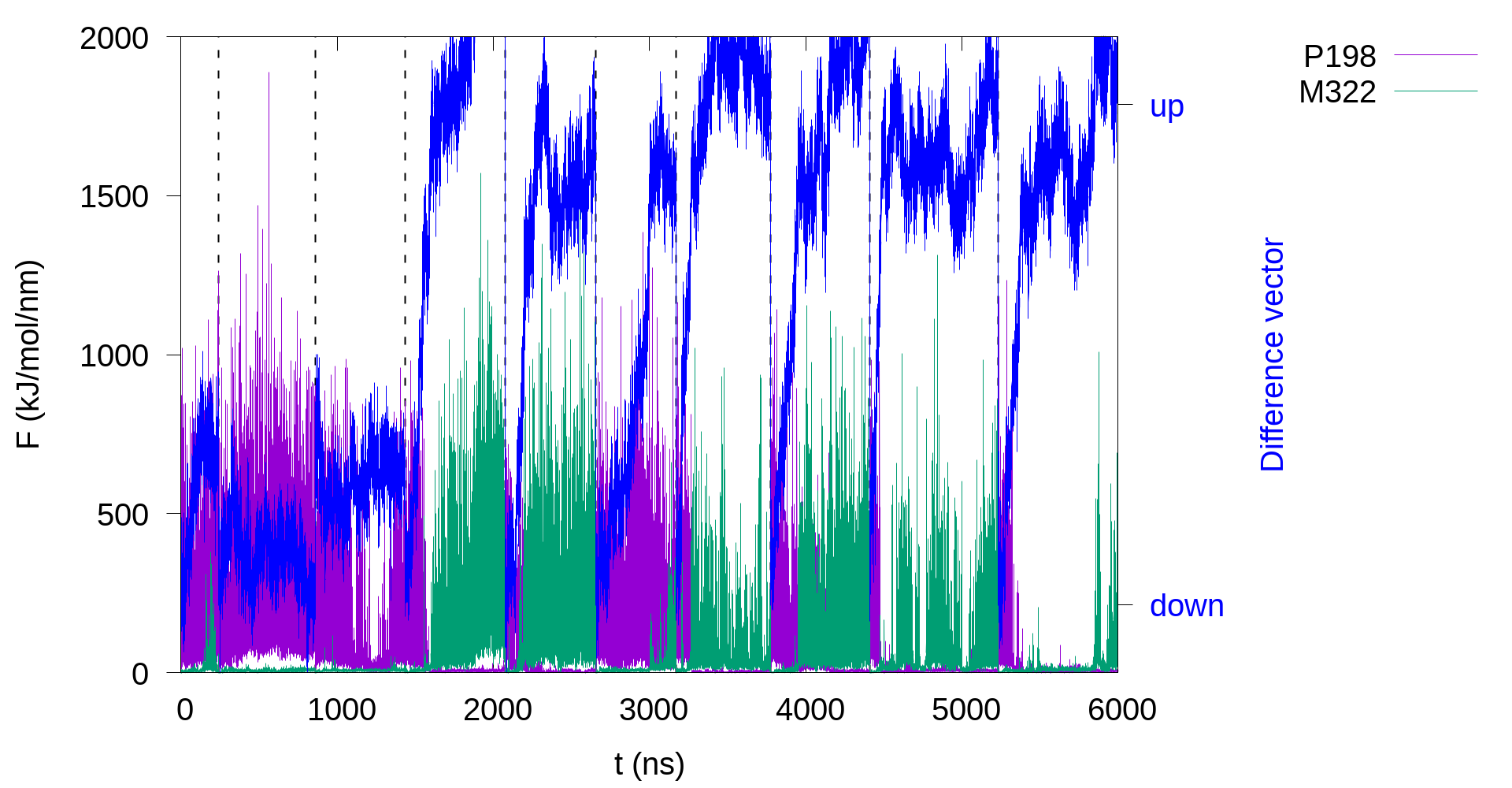
**

**Supplementary Figure S4**: The time dependence of the up-down transition vector (blue) and the membrane-residue force felt by Pro198 (purple) and Met322 (green). All ten trajectories are concatenated, and points where trajectories were spliced together are shown by dashed lines. The data on P198 and M322 refers to the residues from Chain A.

10 20 30 40 50 60

| | | | | |

KCNK1_HUMAN -------------------------------------MLQSLAGSSCVRLVERH------

KCNK7_HUMAN -----------------------------------------MGG---LRPWSR-------

KCNK6_HUMAN ----------------------------------------MRRG----ALLAG-------

**KCNK2_HUMAN MLPSASRERPGYRAGVAAPDLLDPKSAAQ--------NSKPRLSFSTKPTVLASRVESDT**

**KCNKA_HUMAN MFFLYTDFFLSLVAVPAAAPVCQPKSATNGQPPAPAPTPTPRLSISSRATVVA-RMEGTS**

**KCNK4_HUMAN ------------------------------------------------------------**

KCNKG_HUMAN ---------------------------------------------MPSAGLCS-------

KCNKH_HUMAN -------------------------------------MYRPRARAAPEGRVRG-------

KCNK5_HUMAN ------------------------------------------------------------

KCNKC_HUMAN --------------MSSRSPRPPPRRSRR-R----LPRPSCCCCCCRRSHLNE-------

KCNKD_HUMAN --------------MAGRG------------------------FSWGPGHLNE-------

KCNK3_HUMAN --------------------------------------------------MKR-------

KCNK9_HUMAN --------------------------------------------------MKR-------

KCNKF_HUMAN --------------------------------------------------MRR-------

KCNKI_HUMAN -----------------------------------MEVSGHPQARRCCPEALG-------

70 80 90 100 110 120

| | | | | |

KCNK1_HUMAN -------RSAWCFGFLVLGYLLYLVFGAVVFSSVELPYEDLLRQELRKLKRRFLEEH-EC

KCNK7_HUMAN ------------YGLLVVAHLLALGLGAVVFQALEGPPACRLQAELRAELAAFQAEHRAC

KCNK6_HUMAN ------------A---LAAYAAYLVLGALLVARLEGPHEARLRAELETLRAQLLQRS-PC

**KCNK2_HUMAN T---INVMKWKTVSTIFLVVVLYLIIGATVFKALEQPHEISQRTTIVIQKQTFISQH-SC**

**KCNKA_HUMAN QGGLQTVMKWKTVVAIFVVVVVYLVTGGLVFRALEQPFESSQKNTIALEKAEFLRDH-VC**

**KCNK4_HUMAN -------MRSTTLLALLALVLLYLVSGALVFRALEQPHEQQAQRELGEVREKFLRAH-PC**

KCNKG_HUMAN -------CWGGRVLPLLLAYVCYLLLGATIFQLLERQAEAQSRDQFQLEKLRFLENY-TC

KCNKH_HUMAN -------CAVPSTVLLLLAYLAYLALGTGVFWTLEGRAAQDSSRSFQRDKWELLQNF-TC

KCNK5_HUMAN --------MVDRGPLLTSAIIFYLAIGAAIFEVLEEPHWKEAKKNYYTQKLHLLKEF-PC

KCNKC_HUMAN --------DTGRFVLLAALIGLYLVAGATVFSALESPGEAEARARWGATLRNFSAAH--G

KCNKD_HUMAN --------DNARFLLLAALIVLYLLGGAAVFSALELAHERQAKQRWEERLANFSRGH--N

KCNK3_HUMAN --------QNVRTLALIVCTFTYLLVGAAVFDALESEPELIERQRLELRQQELRARY--N

KCNK9_HUMAN --------QNVRTLSLIVCTFTYLLVGAAVFDALESDHEMREEEKLKAEEIRIKGKY--N

KCNKF_HUMAN --------PSVRAAGLVLCTLCYLLVGAAVFDALESEAESGRQRLLVQKRGALRRKF--G

KCNKI_HUMAN --------KLFPGLCFLCFLVTYALVGAVVFSAIEDGQVLVAADDGEFEKFLEELCRILN

130 140 150 160 170 180

| | | | | |

KCNK1_HUMAN LSEQQLEQFLGRVLEASNYGVSVLSNASGNW-----NWDFTSALFFASTVLSTTGYGHTV

KCNK7_HUMAN LPPGALEELLGTALATQAHGVSTLGNSSEGR-----TWDLPSALLFAASILTTTGYGHMA

KCNK6_HUMAN VAAPALDAFVERVLAAGRLGRVVLANASGSANASDPAWDFASALFFASTLITTVGYGYTT

**KCNK2_HUMAN VNSTELDELIQQIVAAINAGIIPLGNTSNQISH----WDLGSSFFFAGTVITTIGFGNIS**

**KCNKA_HUMAN VSPQELETLIQHALDADNAGVSPIGNSSNNSSH----WDLGSAFFFAGTVITTIGYGNIA**

**KCNK4_HUMAN VSDQELGLLIKEVADALGGGADPETNSTSNSSHS--AWDLGSAFFFSGTIITTIGYGNVA**

KCNKG_HUMAN LDQWAMEQFVQVIMEAWVKGVNPKGNSTN---PS--NWDFGSSFFFAGTVVTTIGYGNLA

KCNKH_HUMAN LDRPALDSLIRDVVQAYKNGASLLSNTTS---MG--RWELVGSFFFSVSTITTIGYGNLS

KCNK5_HUMAN LGQEGLDKILEVVSDAAGQGVAITGNQT----FN--NWNWPNAMIFAATVITTIGYGNVA

KCNKC_HUMAN VAEPELRAFLRHYEAALAAGVRADALRPR--------WDFPGAFYFVGTVVSTIGFGMTT

KCNKD_HUMAN LSRDELRGFLRHYEEATRAGIRVDNVRPR--------WDFTGAFYFVGTVVSTIGFGMTT

KCNK3_HUMAN LSQGGYEELERVVLRLK-------PHKAG------VQWRFAGSFYFAITVITTIGYGHAA

KCNK9_HUMAN ISSEDYRQLELVILQSE-------PHRAG------VQWKFAGSFYFAITVITTIGYGHAA

KCNKF_HUMAN FSAEDYRELERLALQAE-------PHRAG------RQWKFPGSFYFAITVITTIGYGHAA

KCNKI_HUMAN CSETVVEDRKQDLQGHLQKVKPQWFNRTT-------HWSFLSSLFFCCTVFSTVGYGYIY

**I198**

190 200 210 220 230 240

| **|**| | | | |

KCNK1_HUMAN PLSDGGKAFCIIYSVIGIPFTLLFLTAVVQRITVHVTRRPVLYFHIR--WG---------

KCNK7_HUMAN PLSPGGKAFCMVYAALGLPAS-LALVATLRHCLLPVLSRPRAWVAVH--WQ---------

KCNK6_HUMAN PLTDAGKAFSIAFALLGVPTTMLLLTASAQRLSLLLTHVPLSWLSMR--WG---------

**KCNK2_HUMAN PRTEGGKIFCIIYALLGIPLFGFLLAGVGDQLGTIFGKGIAKVEDTFIKWN---------**

**KCNKA_HUMAN PSTEGGKIFCILYAIFGIPLFGFLLAGIGDQLGTIFGKSIARVEKVFRKKQ---------**

**KCNK4_HUMAN LRTDAGRLFCIFYALVGIPLFGILLAGVGDRLGSSLRHGIGHIEAIFLKWH---------**

KCNKG_HUMAN PSTEAGQVFCVFYALLGIPLNVIFLNHLGTGL-RAHLAAIERWEDRP-------------

KCNKH_HUMAN PNTMAARLFCIFFALVGIPLNLVVLNRLG----HLMQQGVNHWASRLGGTW---------

KCNK5_HUMAN PKTPAGRLFCVFYGLFGVPLCLTWISALGKFF-GGRAKRLGQFLTKRG------------

KCNKC_HUMAN PATVGGKAFLIAYGLFGCAGTILFFNLFLERIISLLAFIMRACRERQLRRSGLLPATFRR

KCNKD_HUMAN PATVGGKIFLIFYGLVGCSSTILFFNLFLERLITIIAYIMKSCHQRQLRRRGALPQESLK

KCNK3_HUMAN PSTDGGKVFCMFYALLGIPLTLVMFQSLGERINTLVRYLLHRAKKGLG------------

KCNK9_HUMAN PGTDAGKAFCMFYAVLGIPLTLVMFQSLGERMNTFVRYLLKRIKKCCG------------

KCNKF_HUMAN PGTDSGKVFCMFYALLGIPLTLVTFQSLGERLNAVVRRLLLAAKCCLG------------

KCNKI_HUMAN PVTRLGKYLCMLYALFGIPLMFLVLTDTGDILATILSTSYNRFRKFPFFTR---------

250 260 270 280 290 300

| | | | | |

KCNK1_HUMAN ---------FSKQVVAIVHAVLLGFVTVSCFFFIPAAVFSVLEDDWNFLESFYFCFISLS

KCNK7_HUMAN ---------LSPARAALLQAVALGLLVASSFVLLPALVLWGLQGDCSLLGAVYFCFSSLS

KCNK6_HUMAN ---------WDPRRAACWHLVALLGVVVTVCFLVPAVIFAHLEEAWSFLDAFYFCFISLS

**KCNK2_HUMAN ---------VSQTKIRIISTIIFILFGCVLFVALPAIIFKHIEG-WSALDAIYFVVITLT**

**KCNKA_HUMAN ---------VSQTKIRVISTILFILAGCIVFVTIPAVIFKYIEG-WTALESIYFVVVTLT**

**KCNK4_HUMAN ---------VPPELVRVLSAMLFLLIGCLLFVLTPTFVFCYMED-WSKLEAIYFVIVTLT**

KCNKG_HUMAN ---------RRSQVLQVLGLALFLTLGTLVILIFPPMVFSHVEG-WSFSEGFYFAFITLS

KCNKH_HUMAN ---------QDPDKARWLAGSGALLSGLLLFLLLPPLLFSHMEG-WSYTEGFYFAFITLS

KCNK5_HUMAN ---------VSLRKAQITCTVIFIVWGVLVHLVIPPFVFMVTEG-WNYIEGLYYSFITIS

KCNKC_HUMAN GSALSEADSLAGWKPSVYHVLLILGLFAVLLSCCASAMYTSVEG-WDYVDSLYFCFVTFS

KCNKD_HUMAN DAGQCEVDSLAGWKPSVYYVMLILCTASILISCCASAMYTPIEG-WSYFDSLYFCFVAFS

KCNK3_HUMAN ---------MRRADVSMANMVLIGFFSCISTLCIGAAAFSHYEH-WTFFQAYYYCFITLT

KCNK9_HUMAN ---------MRNTDVSMENMVTVGFFSCMGTLCIGAAAFSQCEE-WSFFHAYYYCFITLT

KCNKF_HUMAN ---------LRWTCVSTENLVVAGLLACAATLALGAVAFSHFEG-WTFFHAYYYCFITLT

KCNKI_HUMAN ---------PLLSKWCPKSLFKKKPDPKPADEAVPQIIISAEELPGPKLGTCPSRPSCSM

**M322**

310 320 330 340 **|**  350 360

| | | | **|** | |

KCNK1_HUMAN TIGLGDYVPGEG--YNQKFRELYKIGITCYLLLGLIAMLVVLETFCELHELKKFRKMFYV

KCNK7_HUMAN TIGLEDLLPGRGRSLHPVIYHLGQLALLGYLLLGLLAMLLAVETFSELPQVRAMGKFFRP

KCNK6_HUMAN TIGLGDYVPGEAP--GQPYRALYKVLVTVYLFLGLVAMVLVLQTFRHVSDLHGLTELILL

**KCNK2_HUMAN TIGFGDYVAGG---SDIEYLDFYKPVVWFWILVGLAYFAAVLSMIGDWLRVISKKTKEEV**

**KCNKA_HUMAN TVGFGDFVAGGN--AGINYREWYKPLVWFWILVGLAYFAAVLSMIGDWLRVLSKKTKEEV**

**KCNK4_HUMAN TVGFGDYVAGA---DPRQDSPAYQPLVWFWILLGLAYFASVLTTIGNWLRVVSRRTRAEM**

KCNKG_HUMAN TIGFGDYVVGTD--PSKHYISVYRSLAAIWILLGLAWLALILPLGPLLLH---RCCQLWL

KCNKH_HUMAN TVGFGDYVIGMN--PSQRYPLWYKNMVSLWILFGMAWLALIIKLILSQLETPGRVCSCCH

KCNK5_HUMAN TIGFGDFVAGVN--PSANYHALYRYFVELWIYLGLAWLSLFVNWKVSMFVEVHKAIKKRR

KCNKC_HUMAN TIGFGDLVSSQH--AAYRNQGLYRLGNFLFILLGVCCIYSLFNVISILIKQVLNWMLRKL

KCNKD_HUMAN TIGFGDLVSSQN--AHYESQGLYRFANFVFILMGVCCIYSLFNVISILIKQSLNWILRKM

KCNK3_HUMAN TIGFGDYVALQKD-QALQTQPQYVAFSFVYILTGLTVIGAFLNLVVLRFMTMNAEDEKRD

KCNK9_HUMAN TIGFGDYVALQTK-GALQKKPLYVAFSFMYILVGLTVIGAFLNLVVLRFLTMNSEDERRD

KCNKF_HUMAN TIGFGDFVALQSG-EALQRKLPYVAFSFLYILLGLTVIGAFLNLVVLRFLVASADWPER-

KCNKI_HUMAN ELFERSHALEKQNTLQLPPQAMERSNSCPELVLGRLSYSIISNLDEVGQQVERLDIPLPI

370 380 390 400 410 420

| | | | | |

KCNK1_HUMAN K-----------------------------------------------------------

KCNK7_HUMAN SG----------------------------------------------------------

KCNK6_HUMAN PP---PC-----------------------------------------------------

**KCNK2_HUMAN GEFRAHAAEWTANVTAEFKET---------------------------------------**

**KCNKA_HUMAN GEIKAHAAEWKANVTAEFRET---------------------------------------**

**KCNK4_HUMAN GGLTAQAASWTGTVTARVTQ----------------------------------------**

KCNKG_HUMAN LSLRQGCGAK--------------------------------------------------

KCNKH_HUMAN HSSKEDFKSQSWR-----------------------------------------------

KCNK5_HUMAN RRRKESFESSPHSRKALQVKGSTASKDVNIFSFLSKKEETYNDLIKQIGKKAMKTSGGGE

KCNKC_HUMAN SCRCCARCCPAPGAPLARRN----------------------------------------

KCNKD_HUMAN DSGCCPQCQ--RGLLRSRRN----------------------------------------

KCNK3_HUMAN AEHRALLTRNGQAGGGGGGGSAH-------------------------------------

KCNK9_HUMAN AEERASLAGNR---------NSM-------------------------------------

KCNKF_HUMAN --------AAR-------------------------------------------------

KCNKI_HUMAN IALIVFAYIS------CAA-----------------------------------------

430 440 450 460 470 480

| | | | | |

KCNK1_HUMAN -----KDKDEDQVH----------------------------------IIEHDQLSFSSI

KCNK7_HUMAN -----PVTAEDQGG----------------------------------ILGQDELALSTL

KCNK6_HUMAN -PASFNADEDDRVD----------------------------------ILGPQPESHQQL

**KCNK2_HUMAN -RRRLSVEIYDKFQR------------------ATSIK-------RKLSAELAGNHNQEL**

**KCNKA_HUMAN -RRRLSVEIHDKLQR------------------AATIRSM---ERRRLGLDQRAHSLDML**

**KCNK4_HUMAN -RAGPAAPPPEKEQP------------------LLPPPPC---PAQPLGRPRSPSPPEKA**

KCNKG_HUMAN --AAPGRRPRRGS----------------------------------TAARGVQVTPQDF

KCNKH_HUMAN --QGPDREPESHSPQ------------------QG------------CYPEGPMGIIQHL

KCNK5_HUMAN TGPGPGLGPQGGGLP------------------ALPPSLV---P-LVVYSKNRVPTLEEV

KCNKC_HUMAN -AITPGSRLRRRLAA------------------LGADPAARDSDAEGRRLSGELISMRDL

KCNKD_HUMAN -VVMPGSVRNRCNIS------------------IETDGVA-ESDTDGRRLSGEMISMKDL

KCNK3_HUMAN -TTDTASSTAAAGGGGFRNVYAEVLHFQSMCSCLWYKSREKLQYSIPMIIPRDLSTSDTC

KCNK9_HUMAN -VIHIPEEPRPSRPR----YKADVPDLQSVCSCTCYRSQD---YGGRSVAPQNSFSAKLA

KCNKF_HUMAN ----PPS-PRPPGAP--------------------E-SRG------LWLPRRPARSVGSA

KCNKI_HUMAN -AILPFWETQLDFEN----------------------------AFYFCFVTLTTIGFGDT

490 500 510 520 530 540

| | | | | |

KCNK1_HUMAN TDQAAG------------------------------------------------------

KCNK7_HUMAN PPAAP-------------------------------------------------------

KCNK6_HUMAN SASSH-------------------------------------------------------

**KCNK2_HUMAN TPCRR---------------TLSVN----------------------------------H**

**KCNKA_HUMAN SPEKRSVFAALDTGRFKASSQESINNRPNNLRLKGPEQLNKHGQGASEDNIINKFGSTSR**

**KCNK4_HUMAN QPPSPP------TASALDYPSENLAFID---------------E--S-----------SD**

KCNKG_HUMAN PISKKG-----LGS----------------------------------------------

KCNKH_HUMAN EPSAHA-----AGCGKDS------------------------------------------

KCNK5_HUMAN SQTLRS-----KGHVSRSPDEEAVARAPEDSSPAPEVFMN----------------QLDR

KCNKC_HUMAN TASNKVS-----LALLQKQLSETAN-----------------------------------

KCNKD_HUMAN LAANKAS-----LAILQKQLSEMAN-----------------------------------

KCNK3_HUMAN VEQSHSS-----PGGGGRYSDTPSRR----------------------------------

KCNK9_HUMAN PHYFHSI-----SYKIEEISPSTLKN----------------------------------

KCNKF_HUMAN SVFCH-------VHKLERCARDNLG-----------------------------------

KCNKI_HUMAN VLEHPN-------FFLFFSIYIIVG-----------------------------------

550 560 570 580 590 600

| | | | | |

KCNK1_HUMAN ---------------------------------------------------MKEDQKQNE

KCNK7_HUMAN ---------------------------------------------------------ASG

KCNK6_HUMAN ------------------------------------------------------------

**KCNK2_HUMAN LTSERD--------------------------------------------VLPPLLKTES**

**KCNKA_HUMAN LTKRKNKDLKKTLPEDVQKIYKTFRNYSLDEEKKEEETEKMCNSDNSSTAMLTDCIQQHA**

**KCNK4_HUMAN TQSERG----------------------------------------CPLPRAPRGRRRPN**

KCNKG_HUMAN ------------------------------------------------------------

KCNKH_HUMAN ------------------------------------------------------------

KCNK5_HUMAN ISEECEPWDAQDYHPLIFQDASITFVNTEAGLSDEETSKSSLEDNLAGEESPQQGAEAKA

KCNKC_HUMAN -----------------------------------------------GYPRSVCVNTRQN

KCNKD_HUMAN -----------------------------------------------GCPHQTSTLARDN

KCNK3_HUMAN ------------------------------------------------CLCSGAPRSAIS

KCNK9_HUMAN ------------------------------------------------SLFP----SPIS

KCNKF_HUMAN --------------------------------------------------FS-------P

KCNKI_HUMAN -----------------------------------------------MEIVFIAFKLVQN

610 620 630 640

| | | |

KCNK1_HUMAN PFVATQSSACVDGPANH-------------------------

KCNK7_HUMAN -----QAPAC--------------------------------

KCNK6_HUMAN -----TDYASIPR-----------------------------

**KCNK2_HUMAN IYLNGLTPHCAG----EEIAVIENIK----------------**

**KCNKA_HUMAN ELENGMIPTDTKDREPENNSLLEDRN----------------**

**KCNK4_HUMAN PPRKPVRPRGPG--RPRDKGVPV-------------------**

KCNKG_HUMAN ------------------------------------------

KCNKH_HUMAN ------------------------------------------

KCNK5_HUMAN PLNMGEFPSSSESTFTSTESELSVPYEQLMNEYNKANSPKGT

KCNKC_HUMAN GFSGGVGALGIMNNRLAETSASR-------------------

KCNKD_HUMAN EFSGGVGAFAIMNNRLAETSGDR-------------------

KCNK3_HUMAN SVSTGLHSLSTFRGLMKRRSSV--------------------

KCNK9_HUMAN SISPGLHSFTDHQRLMKRRKSV--------------------

KCNKF_HUMAN PSSPGVVRGGQAPRPGARWKSI--------------------

KCNKI_HUMAN RLIDIYKNVMLFFAKGKFYHLVKK------------------

**Supplementary Figure S5**: Multiple sequence alignment (MSA) of the 15 human K2P channel sequences. We highlight the three mechanosensitive channels: TREK-1 (KCNK2_HUMAN), TREK-2 (KCNKA_HUMAN), and TRAAK (KCNK4_HUMAN) in boldface, and we highlight the two residues mentioned in the main text (M322 and P198) in red. The sequences were obtained from [www.uniprot.org](http://www.uniprot.org) (Accessed: July 15, 2020) and the alignments were performed in UCSF Chimera version 1.7 and default parameters.
